# Switching of cortical Up and Down states: reproduction of the Shu-Hasenstaub-McCormick experiment from a conductance-based model

**DOI:** 10.1186/1471-2202-12-S1-P39

**Published:** 2011-07-18

**Authors:** Arne Weigenand, Thomas Martinetz, Jens Christian Claussen

**Affiliations:** 1 Institute for Neuro- and Bioinformatics, University of Luebeck, Ratzeburger Allee 160, 23562 Luebeck, Germany; 2 Graduate School for Computing in Medicine and Life Science, University of Luebeck, Germany; 3 Sonderforschungsbereich DFG-SFB-654 "Plasticity and Sleep", Campus Luebeck, Germany

## 

Cortical Up- and Down states are a salient feature of mammalian slow wave sleep and contribute predominantely to the low frequency (1Hz and below) delta power of the scalp electroencephalogram (EEG).The role of slow wave oscillations recently raised considerable attention from the observation that slow waves can be electrically stimulated [1,2], and that after such a stimulation, a significant increase in memory consolidation can be observed [1]. Cortical slow-waves are comprised of collective depolarzation (Up) and polarization (Down) phases whereby Up phases show an increased firing rate reminescent of wakefulness [3] and Down states are characterized by comparatively silent levels of neural activity.

A recent experimental demonstration of Up- and Down state switching under electrical stimulation was presented by Shu, Hasenstaub and McCormick [4]. Their experiment showed that in ferret brain slices an Up state could be triggered electrically, as well as that by stimulation during the Up state by an impulse of same polarity, the Up state could be terminated, and the dependence of the Up state duration depending on the impulse amplitude and the

time difference between the two impulses was studied quantitatively.

In a previous study, we have proposed a minimal model mimicking the interplay between recurrent excitation and inhibition controlled by slow adaptive currents [5]. By this quite generic model the experimental time-dependence already can be explained. While the model needs only few parameters, it does not explicitely model neural spiking and it is desired to computationally confirm the approach also from a conductance-based model.

To study stimulation at this level of description, we follow the established conductance-based cortex model by Compte et al. [6] which incorporates a two-compartment (soma and dendrite) membrane potential within a Hodgkin-Huxley type formalism. We use a one-dimensional distance-dependent randomly connected betwork of 256 regular spiking (RS) and 64 fast-spiking (FS) interneurons, an increase of the size did not significantly affect the qualitative behavior.

We observe self-generated Up-Down states in the absence of stimulation in accordance with the detailed study of [6]. If we apply low stimulus intensities, the Up state duration is trivially affected marginally, as in the experiment, and or high stimulus intensities a termination of Up states is enforced, giving two delimiting lines as in [4]. For intermediate stimulus intensites the termination of the Up state depends on the stimulus intensity such that the experiment is confirmed within the measurement precision in [4].
